# DNAscan2: a versatile, scalable, and user-friendly analysis pipeline for human next-generation sequencing data

**DOI:** 10.1093/bioinformatics/btad152

**Published:** 2023-04-03

**Authors:** Heather Marriott, Renata Kabiljo, Ahmad Al Khleifat, Richard J Dobson, Ammar Al-Chalabi, Alfredo Iacoangeli

**Affiliations:** Department of Basic and Clinical Neuroscience, King’s College London, London, United Kingdom; Department of Biostatistics and Health Informatics, King’s College London, London, United Kingdom; Department of Basic and Clinical Neuroscience, King’s College London, London, United Kingdom; Department of Biostatistics and Health Informatics, King’s College London, London, United Kingdom; Department of Basic and Clinical Neuroscience, King’s College London, London, United Kingdom; Department of Biostatistics and Health Informatics, King’s College London, London, United Kingdom; NIHR BRC SLAM NHS Foundation Trust, London, United Kingdom; Institute of Health Informatics and NIHR BRC at University College London Hospitals NHS Foundation Trust, London, United Kingdom; Department of Basic and Clinical Neuroscience, King’s College London, London, United Kingdom; King’s College Hospital, London, United Kingdom; Department of Basic and Clinical Neuroscience, King’s College London, London, United Kingdom; Department of Biostatistics and Health Informatics, King’s College London, London, United Kingdom; NIHR BRC SLAM NHS Foundation Trust, London, United Kingdom

## Abstract

**Summary:**

The current widespread adoption of next-generation sequencing (NGS) in all branches of basic research and clinical genetics fields means that users with highly variable informatics skills, computing facilities and application purposes need to process, analyse, and interpret NGS data. In this landscape, versatility, scalability, and user-friendliness are key characteristics for an NGS analysis software. We developed DNAscan2, a highly flexible, end-to-end pipeline for the analysis of NGS data, which (i) can be used for the detection of multiple variant types, including SNVs, small indels, transposable elements, short tandem repeats, and other large structural variants; (ii) covers all standard steps of NGS analysis, from quality control of raw data and genome alignment to variant calling, annotation, and generation of reports for the interpretation and prioritization of results; (iii) is highly adaptable as it can be deployed and run via either a graphic user interface for non-bioinformaticians and a command line tool for personal computer usage; (iv) is scalable as it can be executed in parallel as a Snakemake workflow, and; (v) is computationally efficient by minimizing RAM and CPU time requirements.

**Availability and implementation:**

DNAscan2 is implemented in Python3 and is available at https://github.com/KHP-Informatics/DNAscanv2.

## 1 Introduction

Thanks to its growing accessibility and affordability, next-generation sequencing (NGS) is now being adopted in all fields of clinical and biomedical genetics. As a consequence, a broad audience of users require flexible and easy-to-use bioinformatics software able to adapt to their informatics proficiency, computing infrastructure, and study objectives. Current publicly available NGS pipelines normally focus on the analysis of specific types of genetic variants, e.g. only SNVs and small indels, or only structural variants, do not cover the whole analysis process (i.e. they are not end-to-end), and are not suitable for users with limited informatics skill (DePristo et al. 2011; Chiang et al. 2015; Collins et al. 2020; Zarate et al. 2020). Although tools that focus on solving some of these factors exist, e.g. being end-to-end (Causey et al. 2018) or user-friendly (Blankenberg et al. 2010), to our knowledge, only commercial bioinformatics solutions which are not accessible to the majority of NGS users, cover all of these aspects (Miller et al. 2015). On such a basis, we developed DNAscan2.

## 2 Results and implementation

DNAscan2 is written in Python3 and is an open-source software tool available to download from GitHub (https://github.com/KHP-Informatics/DNAscanv2). The installation of its software and database dependencies (Supplementary Table S1) can be performed manually, with a bash helper script or via a GUI. An Anaconda (Anaconda Software Distribution (2022), Web: https://anaconda.com) environment file of available binary dependencies is also provided for those who want to install software without package conflicts. The full list of dependencies with their installation specifics are shown in Supplementary Table S2.

### 2.1 New and upgraded features

DNAscan2 presents substantial improvements with respect to DNAscan (Iacoangeli et al. 2019a) in all phases of the analysis (see sections below). Unlike in DNAscan, where users could select one of three modes (fast, normal, and intensive) to tailor the computational requirements to their availability, DNAscan2 implements a single protocol that automatically tailors itself according to the type of variants the user is interested in by default (see Supplementary Fig. S1 and Supplementary Table S2), and it allows the selection of a fast mode which does not perform computationally intensive steps for users with limited RAM and/or CPU time constraints. Descriptions of the benchmarking procedure and detailed results are available in the Supplementary Information.

#### 2.1.1 SNV and indel calling

The Strelka2 small variant caller (Kim et al. 2019) has replaced Freebayes (Garrison and Marth, 2012) and GATK Haplotype Caller (Poplin et al. 2018) for both SNV and indel calling (Supplementary Fig. S1A), as it has a similar performance for SNVs and consistently demonstrates a higher precision and F-measure for indel detection on both NA12878 WES and HG002 WGS samples for both standard calls (Supplementary Figs S2 and 3A) and medically relevant genetic variants in challenging regions (Supplementary Fig. S3B).

#### 2.1.2 Structural variant calling

An enhanced structural variant calling protocol was developed via the addition of Delly (Rausch et al. 2012) to call inversion and deletion variants as well as tandem duplications and translocation events. Delly exhibits a 28–35% higher F-measure for small (50–1000 bp) and medium (1001–10 000 bp) deletions (Supplementary Fig. S4A) on BWA-mem and HISAT2 aligned HG002 WGS reads generated with DNAscan, in addition to a 35% increase in precision for small (101–1000 bp) haplotype-resolved inversion calls (Sudmant et al. 2015) on simulated NA12878 WGS reads (Supplementary Fig. S5A). Furthermore, almost all true positive deletion and inversion calls for both datasets are exclusive to Delly or shared by both Manta and Delly (Supplementary Figs S4B and S5B). This improved calling comes at the expense of increased runtime, with DNAscan2 taking ∼24-30 hours longer to run (Supplementary Table S4). Structural variant calling with Delly is not performed in fast mode.

#### 2.1.3 Transposable element and short tandem repeat discovery

The protocol for the detection of mobile element insertions (Alu, SVA and LINE1) and tandem repeats has been substantially improved with the addition of new state-of-the-art tools. Mobile elements can now be discovered and genotyped via MELT (Gardner et al. 2017) and a genome-wide non-reference short tandem repeat loci profile with details of the motif composition and estimated repeat size of each identified repeat can be generated using ExpansionHunter Denovo (Dolzhenko et al. 2020). Users also have the option to convert the repeat loci into a catalog format compatible with ExpansionHunter (using a conversion script available at https://github.com/francesca-lucas/ehdn-to-eh) to undergo repeat size estimation and genotyping (Supplementary Fig. S1A). Short tandem repeat genotyping is not performed in fast mode.

#### 2.1.4 Variant annotation and report generation

The spectrum of variants that can now be annotated has been extended to include structural and transposable elements (Supplementary Fig. S1A) with the incorporation of AnnotSV (Geoffroy et al. 2018), in addition to known and novel repeat expansions using user-defined ANNOVAR databases. Additionally, an HTML report of variants annotated with AnnotSV produced with the knotAnnotSV program (Geoffroy et al. 2021), and a generalized annotation report giving type, genomic location, overlapping genes and population variant frequency of all identified variants are created for the user’s convenience (Supplementary Fig. S6).

### 2.2 Snakemake and GUI accessibility

To expand the accessibility of DNAscan2, both a graphical user interface (Supplementary Fig. S1B–D) and a Snakemake workflow (available at https://github.com/KHP-Informatics/DNAscanv2_snakemake) have been developed. This renders DNAscan2 available as both an easy-to-use, end-to-end program via its GUI and as a highly scalable command line tool which can be executed on high-performance computing facilities.

### 2.3 Computational performance

DNAscan2 is optimized to minimize the computational resources necessary for its use. The average memory usage in the SNV and indel calling stage for WGS is approximately 1 Gb (Supplementary Table S4, Supplementary Fig. S7); an improvement of 97% compared with DNAscan. DNAscan2 can complete the full protocol, including alignment, full SV calling and annotation, on WGS data in ∼50 hours using 4 CPUs and ∼15 Gb RAM (Supplementary Table S4, Supplementary Figs S7 and S8), which is reduced to ∼20 h when fast mode is implemented, generally within the hardware specifications of a midrange personal computer.

## 3 Conclusions

DNAscan2 adapts to the heterogenic needs of a wide audience that uses NGS data nowadays. It shows potential to be of great value for a broad range of users and applications, e.g. clinical geneticists focusing on disease diagnostics (Iacoangeli et al. 2019b; Supplementary Fig. S9, Supplementary Table S5), as well as biomedical researchers working on large-scale genomic studies.

## Supplementary Material

btad152_Supplementary_Data

## Data Availability

The data presented in this article are available in the article, in its online supplementary material, or by contacting the corresponding author.
