# Supplementary material for: DNAscan2: a versatile, scalable, and user-friendly analysis pipeline for human next-generation sequencing data
Source: Bioinformatics. 2023 Apr 3;39(4):btad152. doi: 10.1093/bioinformatics/btad152 (PMC10112953; doi:10.1093/bioinformatics/btad152)
Supplement: btad152_Supplementary_Data [file btad152_supplementary_data.pdf]

Supplementary Information

12/03/2023

DNAscan2: a versatile, scalable and user-friendly analysis pipeline for human next-generation sequencing data

Heather Marriott[1,2], Renata Kabiljo[1,2], Ahmad Al Khleifat[1], Richard J Dobson[2,3,4], Ammar Al-Chalabi[1,5] and Alfredo Iacoangeli[1,2,3*]

Supplementary Materials and Methods

Datasets

To compare the SNV and indel calling performance of Freebayes, Strelka2 and GATK Haplotype Caller, we used the paired-end Illumina HiSeq4000 WES of NA12878 (NCBI SRA Accession: ERR1905890) and Illumina HiSeqX PCR-free WGS of HG002 (NCBI SRA Accession: SRR14724544). Samples were aligned to the hg19 genome using the `-alignment` flag of DNAscan in both fast and normal mode prior to SNV and indel calling. Alignment is performed by HISAT2 in fast and normal mode, with BWA-mem introduced in the latter for realignment of soft clipped and unaligned reads to improve the detection of small indels and structural variants in downstream calling steps. Structural variant calling performance of Manta and Delly was evaluated using the aligned HG002 reads in addition to simulated paired-end Illumina WGS reads containing the positions of haplotype-resolved deletion and inversion calls of NA12878 (Sudmant *et al.*, 2015), generated and aligned to the hg38 genome using the VISOR package (Bolognini *et al.*, 2020).

For comparing the variant calling and computational power of the new and previous implementations of DNAscan, we utilised 10 UK control WGS samples sequenced as part of the Project MinE amyotrophic lateral sclerosis sequencing consortium (Project MinE ALS Sequencing Consortium, 2018). Genomic DNA from venous blood drawn from patients and controls was isolated using standard methods before DNA integrity was assessed using gel electrophoresis. Samples were sequenced using Illumina's FastTrack Services (San Diego, CA) on the Illumina HiSeq2000 platform. PCR-free library preparation was used to perform 100bp paired-end sequencing, which yielded ~40x coverage across each sample. Sequenced samples were aligned to the hg38 genome using BWA-mem and were provided to us in CRAM format.

Benchmarking of variant callers to include in DNAscan2

It was not necessary for callers associated with every additional functionality to undergo benchmarking as they either demonstrate beneficial value for identifying novel disease-relevant loci i.e. ExpansionHunter Denovo (Rafehi *et al.*, 2019; Fazal *et al.*, 2020), or consistently show high performance in benchmarking studies i.e. MELT for MEI detection (Kosugi *et al.*, 2019; Vendrell-Mir *et al.*, 2019).

SNVs and Indels

The calling performance of Freebayes and GATK HaplotypeCaller was assessed by running DNAscan with the `-variantcalling` flag in fast, normal and intensive modes for the NA12878 WES and HG002 WGS samples. Fast and normal mode uses Freebayes to call SNVs and indels from a coordinate sorted BAM file. Intensive mode employs GATK HaplotypeCaller to call indels from genomic positions (identified from HISAT2 and BWA-mem alignment) that contain at least one read that potentially harbours a deletion or insertion variant. A similar approach was adopted for Strelka2; both SNVs and indels were called from aligned reads generated by fast and normal mode of DNAscan, with the BED file of probable indel variant positions being used to separate output into SNV and indel variant files with the VCFtools `--exclude-bed` and `--bed` flags, analogous to DNAscan intensive mode. For NA12878, Strelka2 was run with the `--exome` flag.

Benchmarking of variant calling performance was performed using hap.py version 0.3.14 (*Haplotype Comparison Tools*, 2021) with the RTG vcfeval engine. An example command is shown below, where the baseline VCF and confident regions BED file correspond to the appropriate true positive callsets used to evaluate the SNV and indel calls:

```
hap.py --false-positives {confident_regions.bed} –-reference hg19.fa –-report-

prefix {outdir} –-engine=vcfeval –-threads {4} {baseline.vcf} {comparison.vcf}
```

The NA12878 WES calls were evaluated against the National Institute of Science and Technology (NIST) with Platinum Genomes phase transfer small variant truth set (version 3.3.2). Confident call regions were defined as the hg19 exome probe locations from the Agilent SureSelect Human All Exon v5 kit (ELID:S04380110). HG002 calls were evaluated with both the truth variants and confident regions of the NIST small variant benchmarking (version 4.2.1) and Genome In A Bottle Challenging Medically-Relevant Genes (GIAB CMRG version 1.00) datasets.

Structural Variants

The structural variant detection power of Manta and Delly was assessed in a similar fashion to that of SNV and indel callers. The HISAT2 and/or BWA-mem aligned BAM files of HG002 and NA12878 WGS samples were processed by Manta as per the old DNAscan implementation, with the same approach adopted for Delly as to obtain a comparable dataset.

Calls were then evaluated using Truvari version 2.2.1 (*spiralgenetics/truvari*, 2021) using the following command:

```
truvari bench -b {baseline.vcf} -c {comparison.vcf} -f {reference.fasta} -o

{outdir} --includebed {confident_regions.bed}
```

For HG002, the performance of Delly and Manta on detecting deletions was evaluated using the variant calls and confident regions of the GIAB CMRG deletion SV truth set (version 0.6). Deletion and inversion calls of NA12878 were evaluated using calls from the 1000 Genomes structural variant map (Sudmant *et al.*, 2015) as the comparison VCF. To further compare the obtained structural variant calls, the `--sizemin` and `--sizemax` flags were supplied to Truvari. Parameters were defined as 50-100, 101-1000, 1001-10000 and 10000-50000bp (the default `--sizemax` parameter of Truvari).

Comparison between DNAscan and DNAscan2

Once DNAscan2 had been finalised and implemented, both versions of DNAscan were run with the `-variantcalling`, `-SV`, `-expansion`, `-annotation` and `-resultsreport` flags on each of the 10 Project MinE UK control samples. DNAscan2 was additionally run with the `-MEI` and `-STR` flags.

Performance Metrics

Variant Calling Performance

Both SNV/indel and structural variant calls were evaluated using precision, recall and F-measure metrics calculated by hap.py and Truvari, where precision is $\frac{True\ Positives}{(True\ Positives + False\ Negatives)}$, recall is $\frac{True\ Positives}{(True\ Positives + False\ Positives)}$ and F1 score is $\frac{True\ Positives}{True\ Positives + \frac{1}{2}(False\ Positives + False\ Negatives)}$.

Where possible, calling performance of DNAscan and DNAscan2 was assessed by averaging the number of SNV/indel, SV, repeat expansion and MEI calls and STR loci obtained for all Project MinE samples. SV and MEI calls were also separated into their respective subclasses i.e. deletions, insertions, inversions, duplications, Alu, SVA, LINE1. Additionally, the number and percentage of filtered synonymous and non-synonymous SNV variants was acquired from the classification given by refGene annotation.

Computational Performance

Computational efficiency of the SNV/indel and structural variant callers was assessed by obtaining the wall and CPU time (in hh:mm:ss format) and RAM usage (maximum resident set size in gigabytes) for HG002 and NA12878 samples from the SLURM `sacct` command `--format “Elapsed,CPUTime,MaxRSS”`. The same approach was applied for the comparison of DNAscan and DNAscan2, albeit with time and memory usage being averaged across the 10 samples, and estimated disk read and write usage obtained with addition of `“MaxDiskRead,MaxDiskWrite”` to the above `sacct` command.

Storage Requirements of DNAscan2

Total storage required for installation of dependencies, databases and references with DNAscan2 for both hg19 and hg38 genome versions was calculated with the `du -sh` shell command.

Hardware

All tests were performed on an Intel Xeon E5-2670 2.6GHz processor with 4 CPUs and 16Gb RAM (in line with the standard computational requirements of DNAscan), except for processes which involved Freebayes, which ran with 4 CPUs and 64Gb RAM.

Availability of Data and Materials

The variant call file containing haplotype-resolved deletion and inversion calls of NA12878 is available at:

http://ftp.1000genomes.ebi.ac.uk/vol1/ftp/phase3/integrated_sv_map/supporting/GRCh38_positions/ALL.wgs.integrated_sv_map_v1_GRCh38.20130502.svs.genotypes.vcf.gz

The NA12878 NIST with Platinum Genomes phase transfer small variant callset (version 3.3.2) is available at:

https://ftp-trace.ncbi.nlm.nih.gov/giab/ftp/release/NA12878_HG001/latest/GRCh37/HG001_GRCh37_GIAB_highconf_CG-IllFB-IllGATKHC-Ion-10X-SOLID_CHROM1-X_v.3.3.2_highconf_PGandRTGphasetransfer.vcf.gz

Probe regions used for the whole exome sequencing of NA12878 are available from the Agilent SureDesign Dashboard (https://earray.chem.agilent.com/suredesign/). Account registration and sign-in is required to access the regions file from the Agilent catalogue (S04380110_Regions.bed).

The HG002 NIST small variant benchmarking callset containing variant calls and confident regions files (version 4.2.1) are available at the following directory:

https://ftp-trace.ncbi.nlm.nih.gov/giab/ftp/release/AshkenazimTrio/HG002_NA24385_son/NISTv4.2.1/GRCh37/

The HG002 GIAB CMRG small variant calls and confident regions files (version 1.00) are available at the following directory:

https://ftp-trace.ncbi.nlm.nih.gov/ReferenceSamples/giab/release/AshkenazimTrio/HG002_NA24385_son/CMRG_v1.00/GRCh37/SmallVariant/

Supplementary Figures

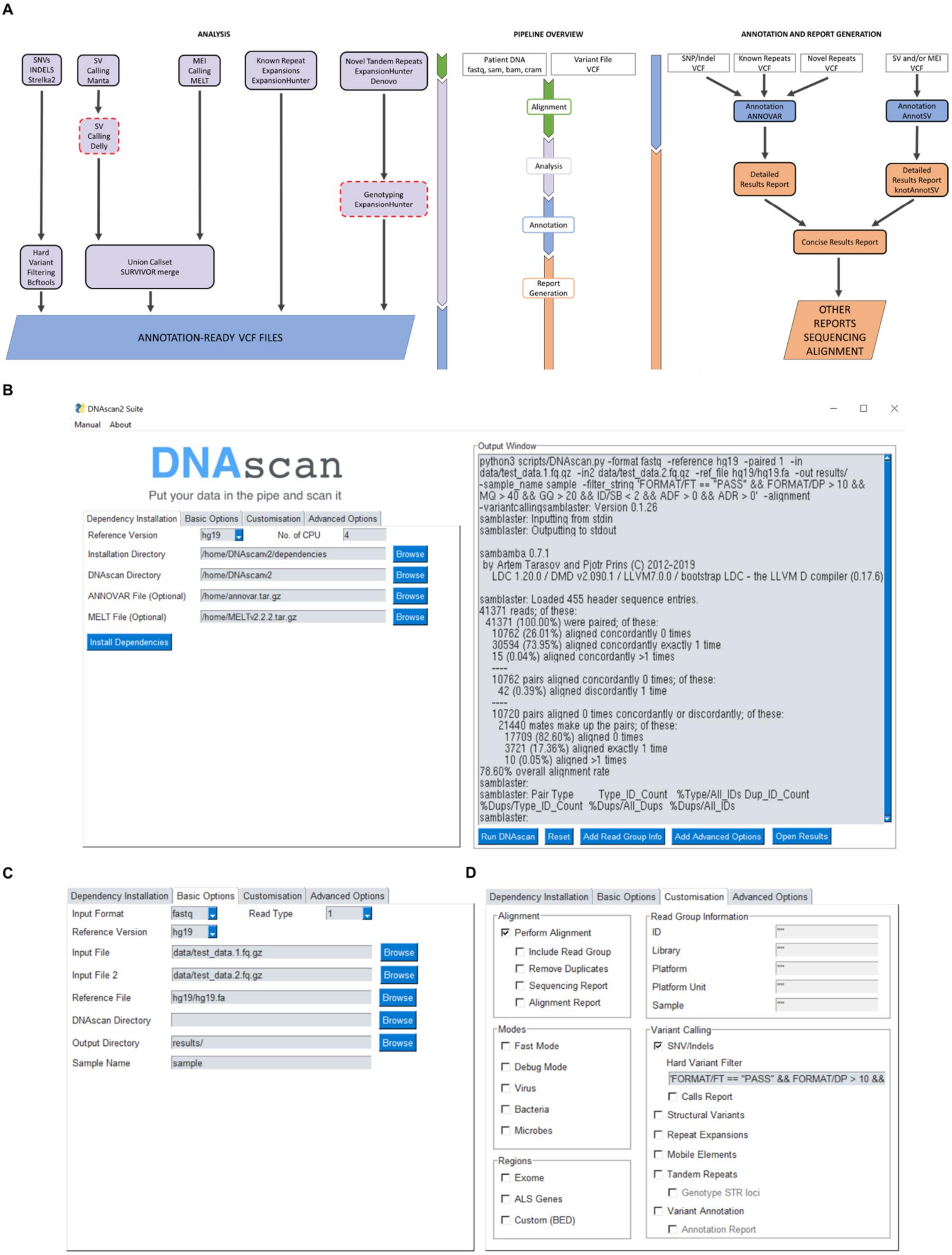

Supplementary Fig. 1. Panel A: Overview of the DNAscan2 pipeline, which consists of four main stages: Alignment, Analysis, Annotation and Report Generation, following input of patient DNA in several different formats. Steps in red dotted lines are removed if the '-fast_mode' flag is set, which massively increases computational efficiency for those with limited computing resources. Panel B: Screengrab of the main DNAscan2 GUI window with an example alignment and variant calling output. Panel C: 'Basic Options' tab displaying the default settings. Panel D: 'Customisation' tab.

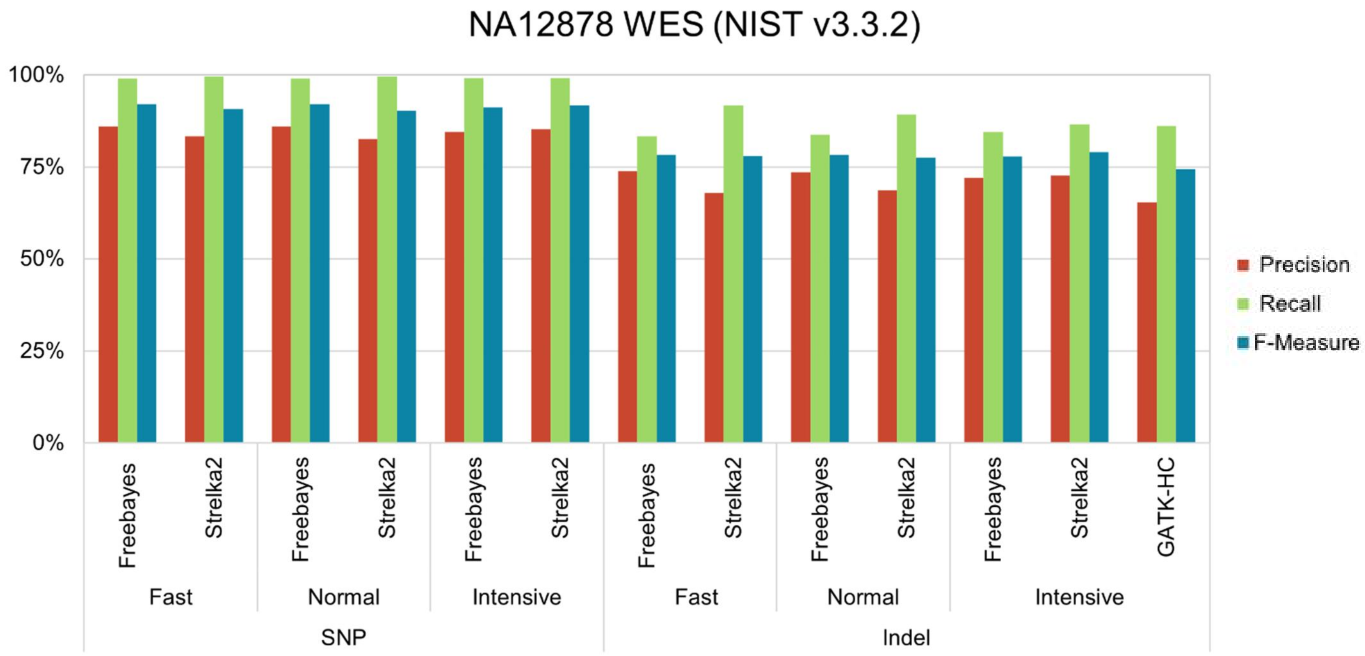

Supplementary Fig. 2. SNV and indel calling performance of Freebayes, Strelka2 and GATK HaplotypeCaller on the whole exome sequencing of NA12878, against the NIST small variant benchmarking callset (version 3.3.2). DNAscan was run in fast, normal and intensive mode.

A

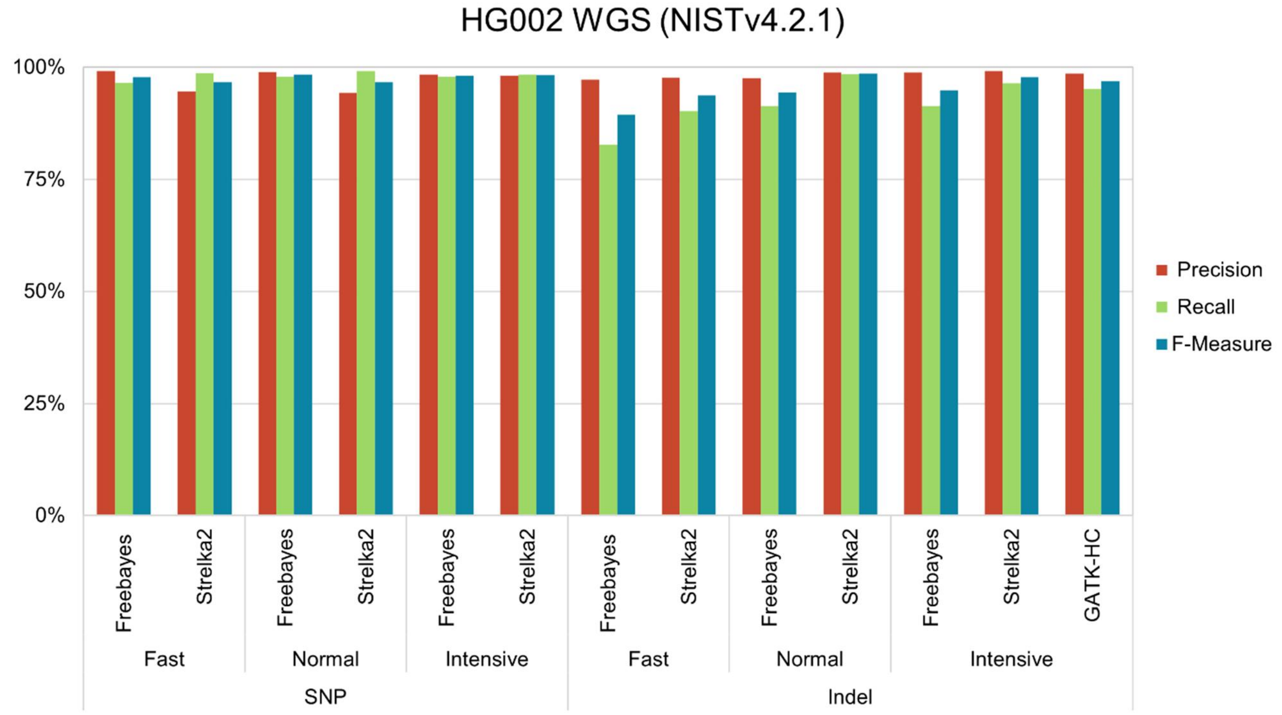

B

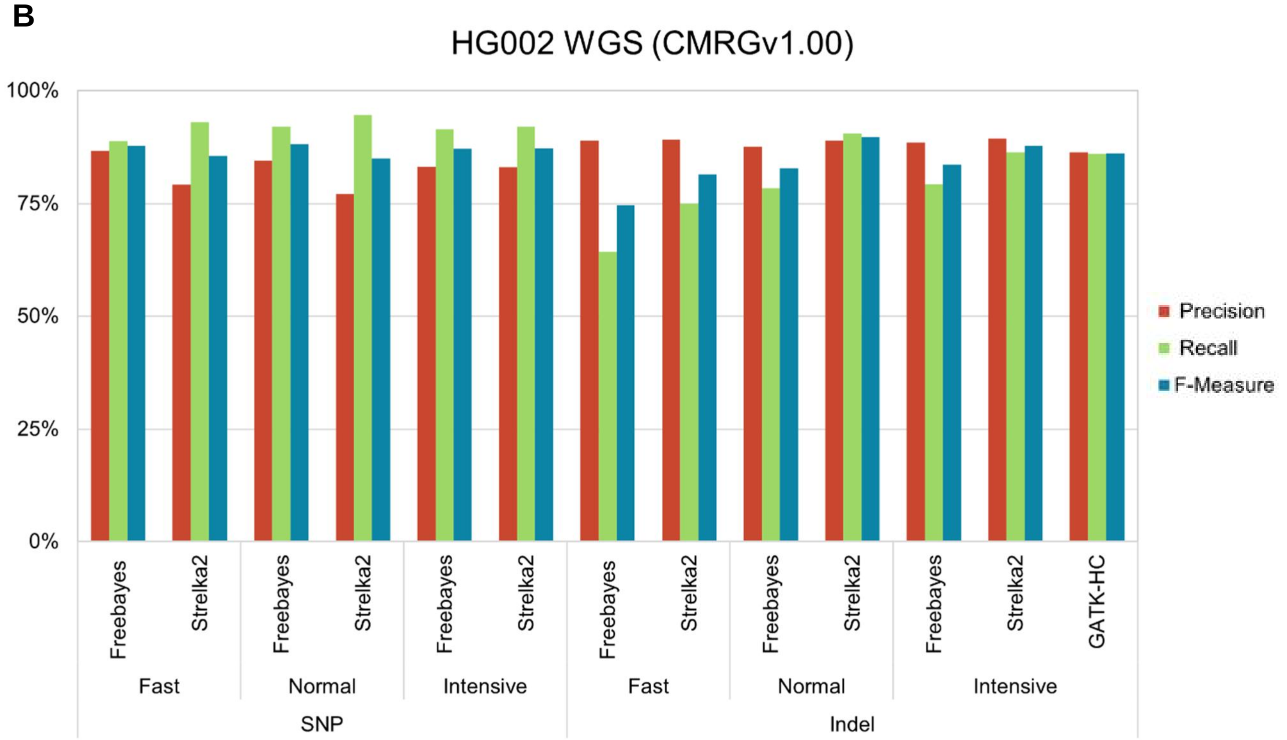

Supplementary Fig. 3. SNV and indel calling performance of Freebayes, Strelka2 and GATK HaplotypeCaller on the whole genome sequencing of HG002, against the **A.** NIST small variant benchmarking callset (version 4.2.1), and the **B.** GIAB CMRG structural variant callset (version 1.00). DNAscan was run in fast, normal and intensive mode.

A

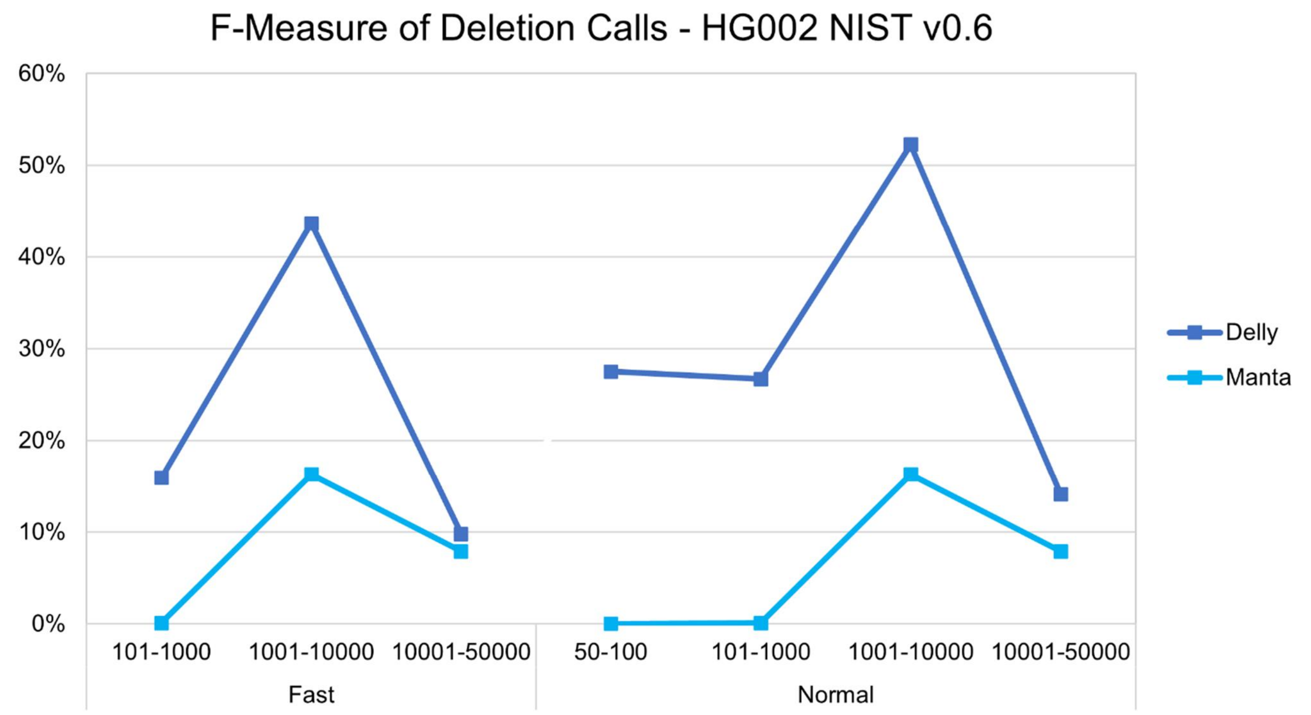

B

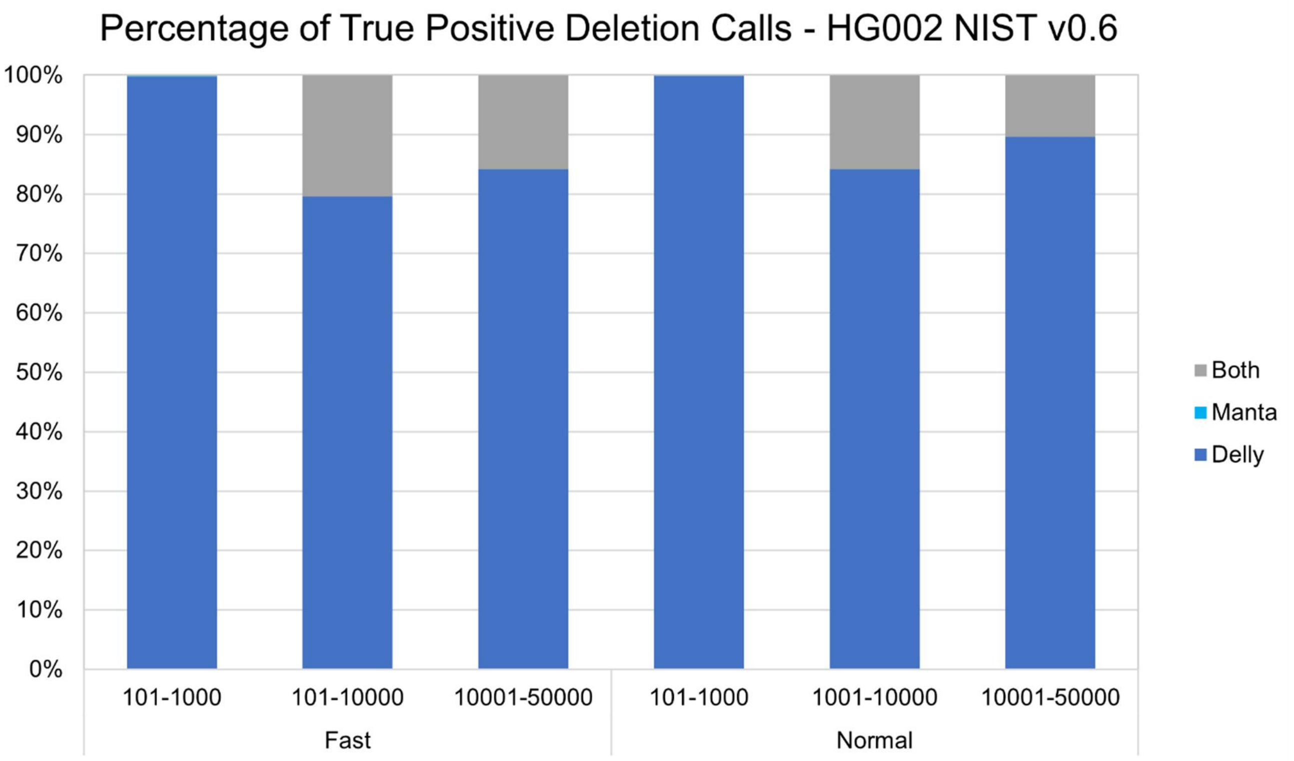

Supplementary Fig. 4. Deletion SV calling performance of Manta and Delly on the whole genome sequencing of HG002 against the NIST SV benchmarking callset (version 0.6). Fast and normal modes refer to the alignment specifics as defined by DNAscan. Fast: HISAT2 alignment. Normal: HISAT2 alignment and BWA-mem realignment of soft-clipped and/or unaligned reads. **A.** F-Measure of calls, and **B.** Percentage of true positive calls shared by or exclusive to Manta and Delly, in fast and normal mode for multiple deletion variant sizes.

A

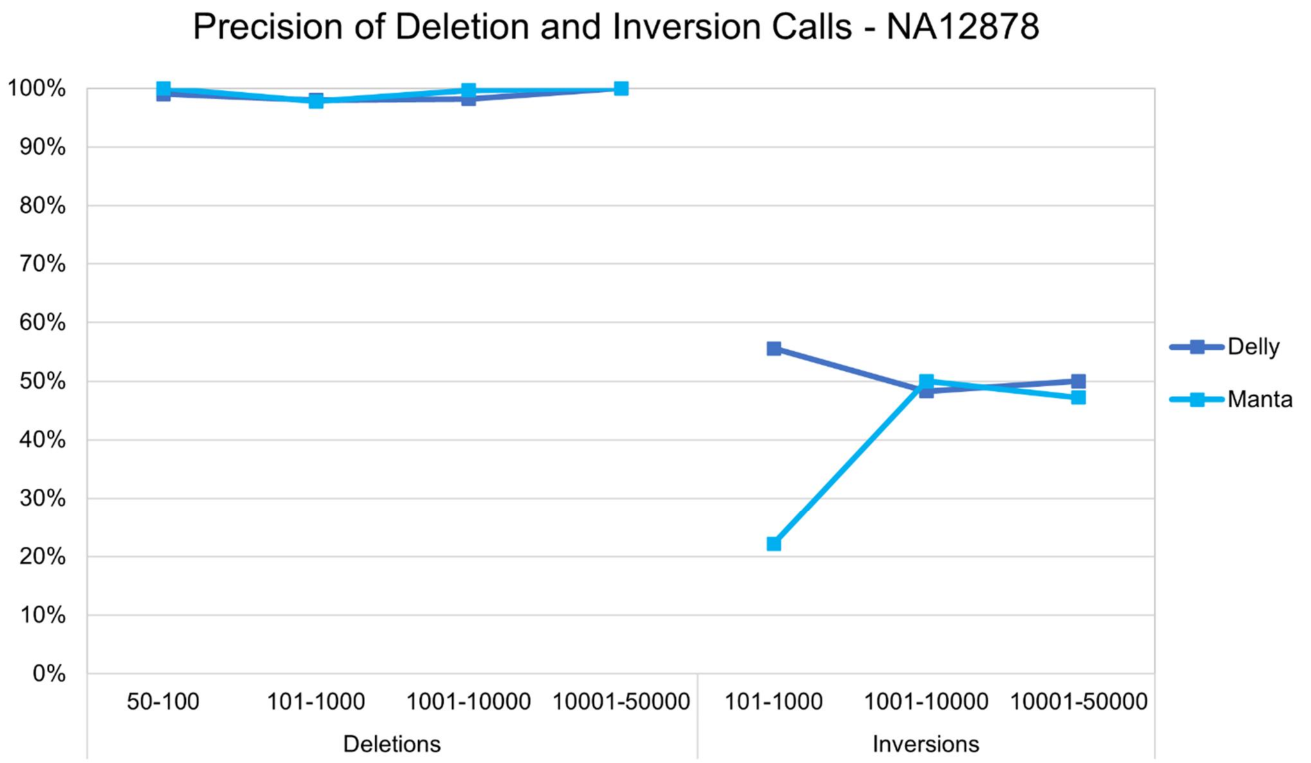

B

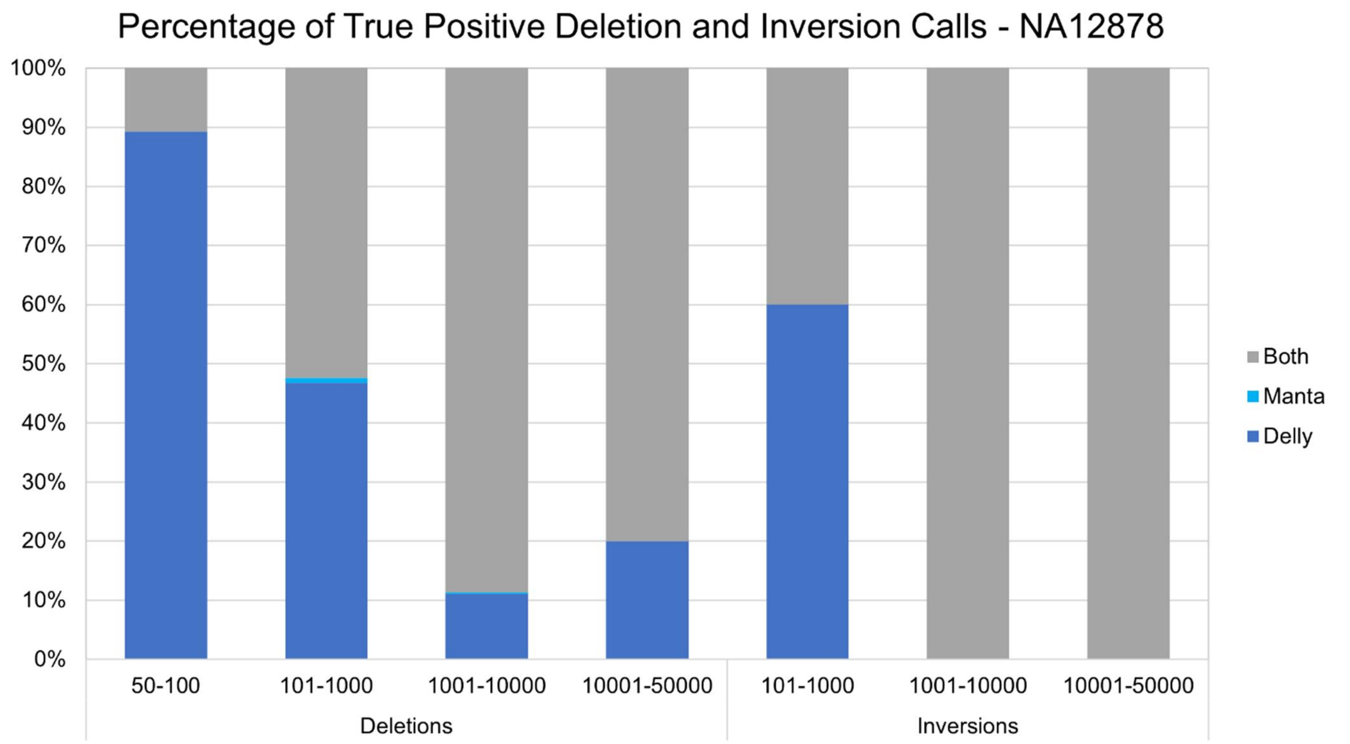

Supplementary Fig. 5. Deletion and inversion SV calling performance of Manta and Delly on simulated whole genome sequencing reads of NA12878 generated with VISOR. **A.** Precision of calls, and **B.** Percentage of true positive calls shared by or exclusive to Manta and Delly, for multiple deletion and inversion variant sizes.

AnnotSV ID	ACMG class	SV type	Annotation mode	Gene name	Location	OMIM ID	Exomiser score	Overlapped regulatory elements	Pathogenic SV	Number of pathogenic SNV/indel overlapped	Benign SV	Left breakpoint annotations	Right breakpoint annotations
21_10441055_10441056_ALU_1	NA	ALU	full	BAGE; BAGE2; BAGE3; BAGE4; [...4genes]	.	.	NA	TPTE; ------ BAGE2;	.	.	DDD:55340	High Signal Region	High Signal Region
21_10441055_10441056_ALU_1	full=NA	ALU	split	BAGE4	intron2-intron2	.	NA	.	.	.	DDD:55340	.	.
21_10441055_10441056_ALU_1	full=NA	ALU	split	BAGE	intron3-intron3	.	NA	.	.	.	DDD:55340	.	.
21_10441055_10441056_ALU_1	full=NA	ALU	split	BAGE3	intron2-intron2	.	NA	.	.	.	DDD:55340	.	.
21_10441055_10441056_ALU_1	full=NA	ALU	split	BAGE2	intron2-intron2	.	NA	.	.	.	DDD:55340	.	.
13_21320641_21320642_ALU_1	NA	ALU	full	GRK6P1; LINC00539; MIPEPP3; [...3genes]	.	.	NA	.	.	.	1000g	.	.
13_21320641_21320642_ALU_1	full=NA	ALU	split	GRK6P1	exon1-exon1	.	NA	.	.	.	1000g	.	.
13_21320641_21320642_ALU_1	full=NA	ALU	split	MIPEPP3	intron2-intron2	.	NA	.	.	.	1000g	.	.
13_21320641_21320642_ALU_1	full=NA	ALU	split	LINC00539	intron2-intron2	.	NA	.	.	.	1000g	.	.
12_11125814_11125815_ALU_1	NA	ALU	full	PRH1; PRH1-PRR4; PRH1-TAS2R14; [...3genes]	.	.	NA	.	.	.	DDD:37842	.	.
12_11125814_11125815_ALU_1	full=NA	ALU	split	PRH1	intron1-intron1	.	NA	.	.	.	DDD:37842	.	.
12_11125814_11125815_ALU_1	full=NA	ALU	split	PRH1-TAS2R14	intron1-intron1	.	NA	.	.	.	DDD:37842	.	.
12_11125814_11125815_ALU_1	full=NA	ALU	split	PRH1-PRR4	intron1-intron1	.	NA	.	.	.	DDD:37842	.	.
12_11066593_11066594_LINE1_1	NA	LINE1	full	PRH1; PRH1-PRR4; PRH1-TAS2R14; [...3genes]	.	.	NA	.	.	.	DDD:37842; IMH; dgv454e212; gnomAD-SV_v2.1_DU[...]	.	.
12_11066593_11066594_LINE1_1	full=NA	LINE1	split	PRH1	intron1-intron1	.	NA	.	.	.	DDD:37842; IMH; dgv454e212; gnomAD-SV_v2.1_DU[...]	.	.
12_11066593_11066594_LINE1_1	full=NA	LINE1	split	PRH1-TAS2R14	intron1-intron1	.	NA	.	.	.	DDD:37842; IMH; dgv454e212; gnomAD-SV_v2.1_DU[...]	.	.
12_11066593_11066594_LINE1_1	full=NA	LINE1	split	PRH1-PRR4	intron1-intron1	.	NA	.	.	.	DDD:37842; IMH; dgv454e212; gnomAD-SV_v2.1_DU[...]	.	.
18_55478844_55478845_ALU_1	NA	ALU	full	TCF4; TCF4-AS1; [...2genes]	.	602228; 602272;	NA	MIR4529;	.	.	1000g	.	.
18_55478844_55478845_ALU_1	full=NA	ALU	split	TCF4	intron1-intron1	602228; 602272;	NA	.	.	.	1000g	.	.
18_55478844_55478845_ALU_1	full=NA	ALU	split	TCF4-AS1	intron1-intron1	.	NA	.	.	.	1000g	.	.
12_106497183_106497184_ALU_1	NA	ALU	full	LOC100287944; POLR3B; [...2genes]	.	614366;	NA	.	.	.	1000g	.	.
12_106497183_106497184_ALU_1	full=NA	ALU	split	POLR3B	intron25-intron25	614366;	NA	.	.	.	1000g	.	.
12_106497183_106497184_ALU_1	full=NA	ALU	split	LOC100287944	intron3-intron3	.	NA	.	.	.	1000g	.	.
3_45501170_45501171_ALU_1	NA	ALU	full	LARS2; LARS2-AS1; [...2genes]	.	604544;	NA	.	.	.	1000g	.	.
3_45501170_45501171_ALU_1	full=NA	ALU	split	LARS2	intron14-intron14	604544;	NA	.	.	.	1000g	.	.
3_45501170_45501171_ALU_1	full=NA	ALU	split	LARS2-AS1	intron2-intron2	.	NA	.	.	.	1000g	.	.

Supplementary Fig. 6. Screengrab of the transposable element report generated by DNAscan2 using knotAnnotSV for 1 Project MinE control sample.

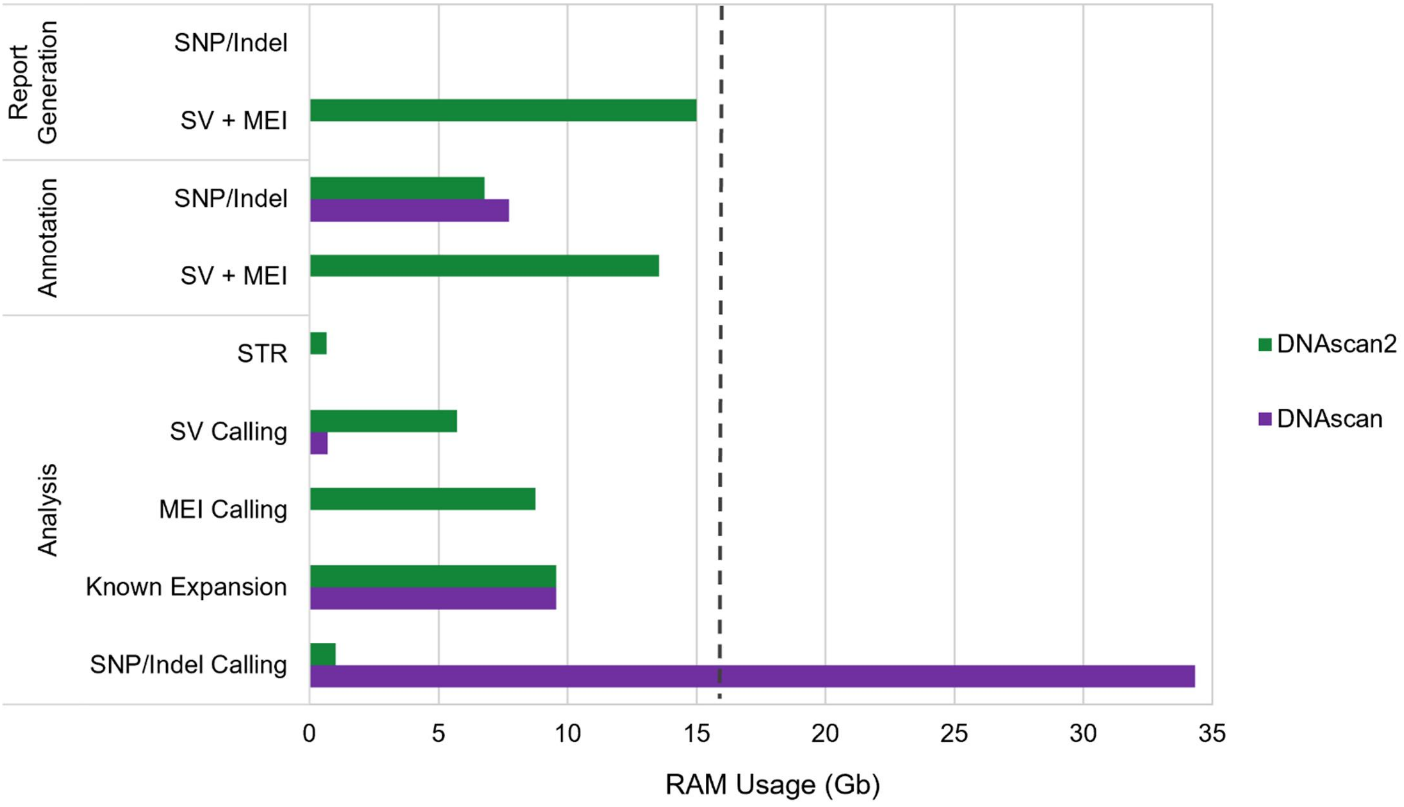

Supplementary Fig. 7. Comparison of average memory usage (in gigabytes) between DNAscan and DNAscan2 for each step following alignment, using whole genomes of 10 Project MinE control samples. The steps were categorised by the main stage of the workflow (Analysis, Annotation, Report Generation). The dotted line represents the average RAM of a standard midrange personal computer.

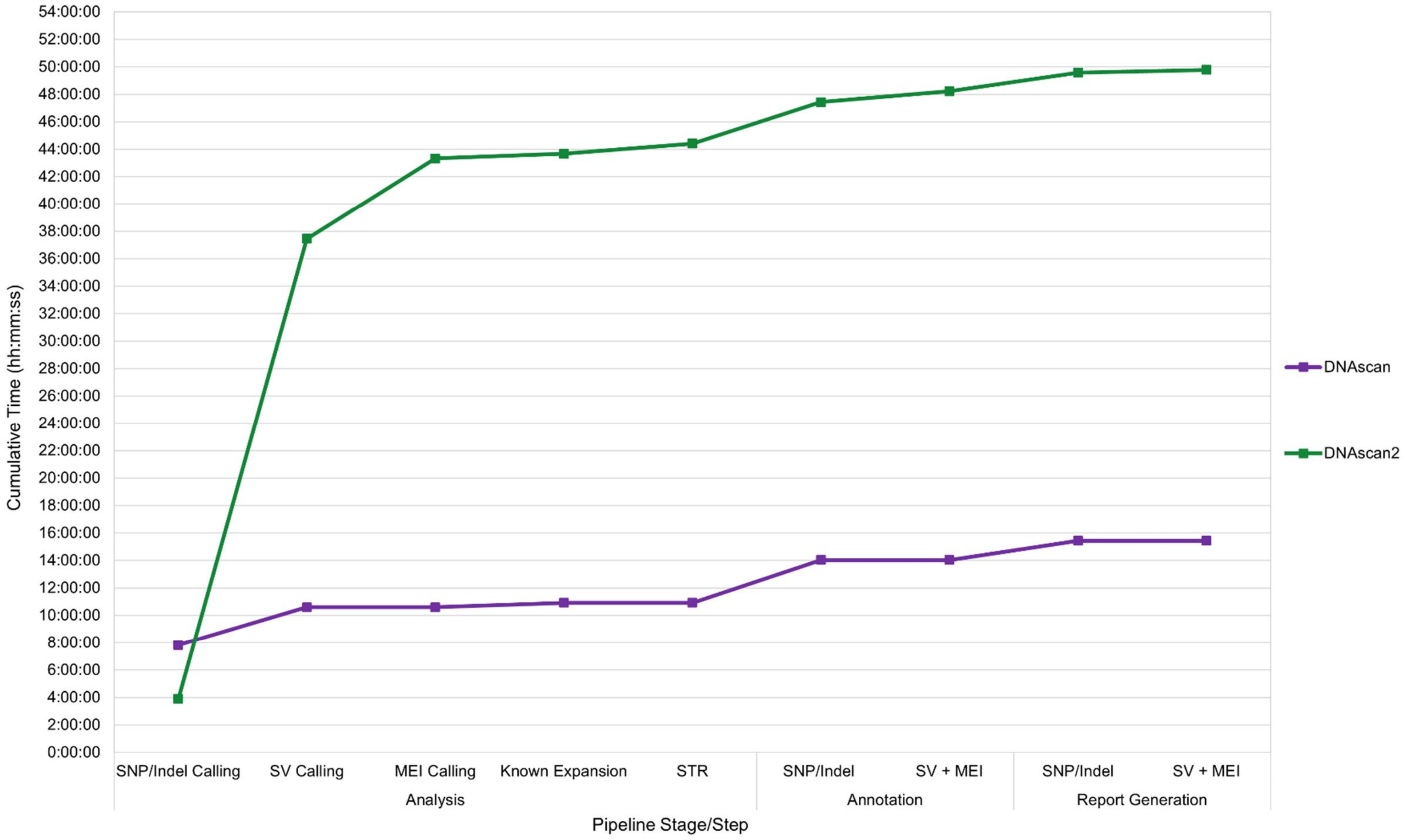

Supplementary Fig. 8. Average cumulative elapsed time (in hours:minutes:seconds format) between DNAscan and DNAscan2 for each step following alignment, using whole genomes of 10 Project MinE control samples. The steps were categorised by the main stage of the workflow (Analysis, Annotation, Report Generation). Separate performance metrics for each stage are listed in Supplementary Table 5.

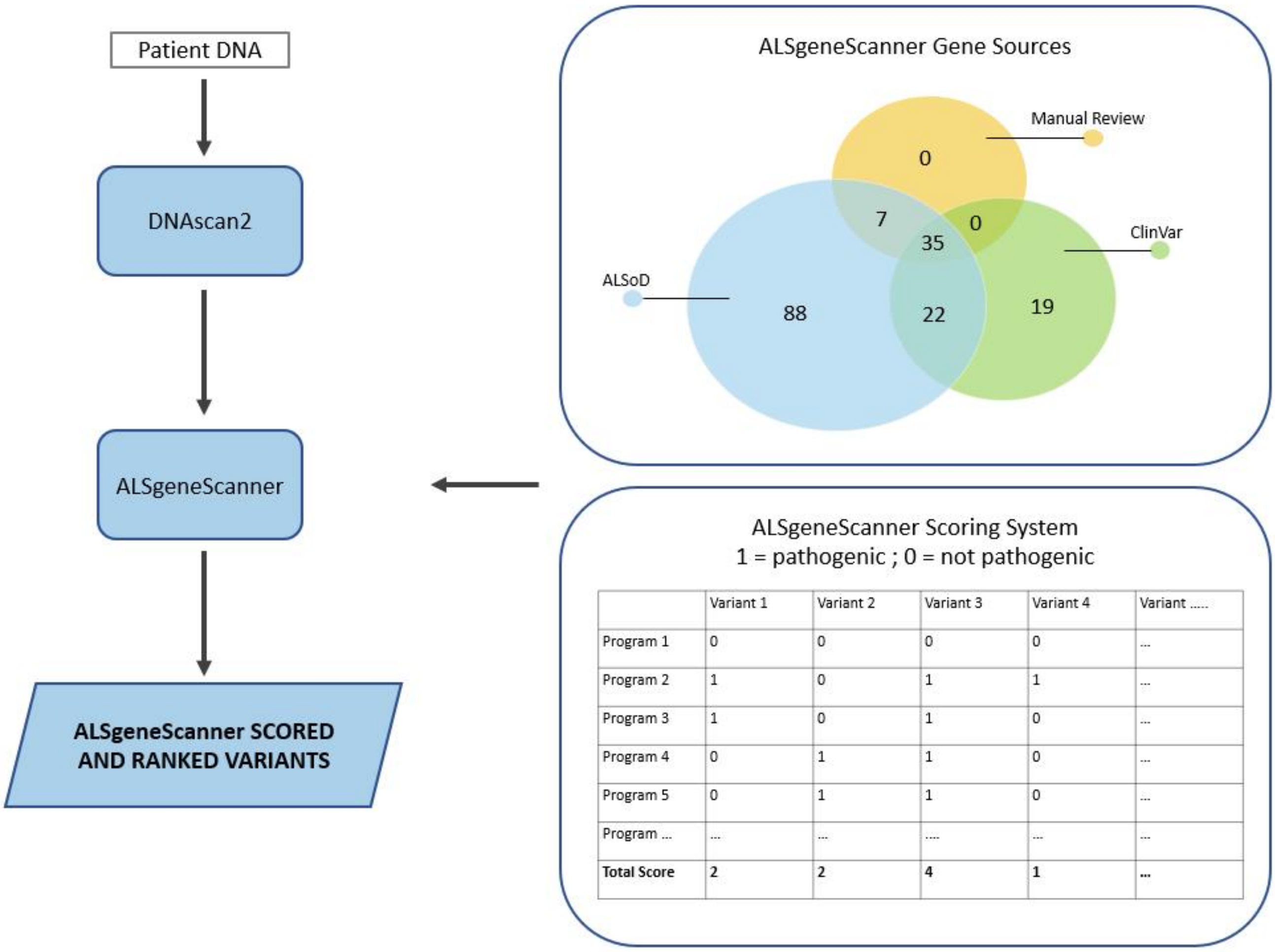

Supplementary Fig. 9. The ALSgeneScanner scoring module of DNAscan2. After the main DNAscan pipeline has completed, ALSgeneScanner takes SNV and indel annotation files as input and keeps only the variants whose genes are identified to be associated with ALS (171 in total, from three different sources). Afterwards, each of these filtered variants are scored based on the pathogenicity prediction assigned by each of the 13 prediction databases (SIFT, Polyphen-2 HDIV, PolyPhen-2 HVAR, LRT, MutationTaster, MutationAssessor, FATHMM, PROVEAN, FATHMM-mkl coding, MetaSVM, CADD and Intervar) and then ranked based on the total cumulative score from all programs.

Supplementary Tables

Dependency Category	Required Storage (Gb)	
	**hg19**	**hg38**
References		
Reference Genome and Index	3.16	3.27
BWA-mem Index	5.42	5.62
HISAT2 Index	4.37	4.66
Databases and Catalogues		
ExpansionHunter Variant Catalogue	< 0.01	< 0.01
ANNOVAR Databases*	91.34	91.67
AnnotSV Databases**	3.31	3.31
Tools		
Conda Installation	0.10	0.10
Conda-Installed Tools	1.50	1.50
Manually Installed Tools	12.01	12.01
Additional Disk Space*	20.00	20.00
**Total free space needed:**	**141.21**	**142.14**

Supplementary Table 1. Storage requirements of all dependencies necessary to run DNAscan2, divided by reference version. *Default DNAscan2 ANNOVAR databases (refGene, dbnsfp33a, clinvar_20210501, intervar_20180118, avsnp147, exac03,1000g2015aug, gnomad211_genome) were used. **AnnotSV was only run using databases that do not require a commercial licence to download. ***This refers to the additional overhead storage space that MELT requires to temporarily hold intermediate files.

Pipeline Stage	Tool	Tool Version	Installation Method	Reference
Alignment				
	BWA-mem	0.7.17	Conda	(Li, 2013)
	HISAT2	2.2.1	Conda	(Kim *et al.*, 2019)
	Samblaster	0.1.26	Conda	(Faust and Hall, 2014)
	Sambamba	0.7.1	Conda	(Tarasov *et al.*, 2015)
Analysis				
	Strelka2	2.9.10	Manual Download	(Kim *et al.*, 2018)
	Manta	1.6.0	Manual Download	(Chen *et al.*, 2016)
	Delly	0.8.3	Conda	(Rausch *et al.*, 2012)
	MELT	2.2.2	Manual Registration and Download	(Gardner *et al.*, 2017)
	ExpansionHunter	3.2.2	Conda	(Dolzhenko *et al.*, 2019)
	ExpansionHunter Denovo	0.9.0	Manual Download	(Dolzhenko *et al.*, 2020)
	SURVIVOR	1.0.7	Manual Download	(Jeffares *et al.*, 2017)
Annotation				
	ANNOVAR	08/06/2019	Manual Registration and Download	(Wang *et al.*, 2010)

	AnnotSV	3.0.9	Manual Download	(Geoffroy *et al.*, 2018)
Report Generation				
	FastQC	0.11.9	Conda	(*FastQC*, no date)
	MultiQC	1.10.1	Conda	(Ewels *et al.*, 2016)
	knotAnnotSV	1.0.0	Manual Download	(Geoffroy *et al.*, 2021)
General				
	Miniconda	4.10.3	Manual Download	(Anaconda Software Distribution. (2022))
	Perl	5.26.2	Conda	
	Python	3.8	Conda	
	Biopython	1.78	Conda	(Cock *et al.*, 2009)
	Pysam	0.16.0.1	Conda	(*Pysam*, 2021)
	SAMtools	1.9	Conda	(Li *et al.*, 2009)
	BEDTools	2.25.0	Conda	(Quinlan and Hall, 2010)
	BCFtools	1.9	Conda	(Li, 2011)
	VCFtools	0.1.16	Conda	(Danecek *et al.*, 2011)
	PySimpleGUI*	4.40.0	Conda	(*PySimpleGUI*, no date)

	Snakemake**	≥ 5.32.1	Conda	(Mölder *et al.*, 2021)

Supplementary Table 2. List of tools and corresponding versions that DNAscan2 requires for each pipeline stage. 'General' refers to tools which are necessary for the basic execution of DNAscan2. The 'Installation Method' column lists the easiest way to obtain the software to avoid software version clashes. *This is only required if you want to run DNAscan2 as a standalone application and not as a command-line tool. **This is only required if you want to deploy DNAscan2 on a high performance computing facility or execute the workflow in a multi-sample parallel fashion.

	DNAscan	DNAscan2
SNV/Indel Calling		
- Total called variants	29192123	30000110
- Total called and filtered variants	4285989	4611498
- Number of filtered non-synonymous variants	10165 (0.237%)	10727 (0.250%)
- Number of filtered synonymous variants	21304 (0.497%)	22285 (0.483%)
Structural Variant Calling		
- Total called variants	28831	56970
· Deletions	8478	16069
· Insertions	6455	6189
· Inversions	3410	7280
· Duplications	4031	9285
· Translocations	6457	18147
MEI Calling		
- Total called variants	N/A	1694
· ALU	N/A	1287
· SVA	N/A	148
· LINE1	N/A	259
Expansion/Repeat Scanning		
- Total called and genotyped expansions	37	37
- Total Identified STR loci	N/A	3043

Supplementary Table 3. Comparison of the variant calling power of DNAscan and DNAscan2 for several variant classes. ‘N/A’ represents tasks that could not be performed in DNAscan due to lack of available software. Synonymous and non-synonymous SNV and indel variants were obtained from the corresponding refGene annotation classification. Values here denote the average number of variant types identified using whole genomes of 10 Project MinE control samples.

	Pipeline Stage	Pipeline Step	Elapsed Time (hh:mm:ss)	CPU Time (hh:mm:ss)	RAM Usage (Gb)	MaxDiskRead	MaxDiskWrite
**DNAscan**	Analysis	SNV/Indel Calling	07:48:52	32:10:31	34.34	144046	71073
		SV Calling	02:46:41	11:06:44	0.69	827571	338
		MEI Calling	N/A	N/A	N/A	N/A	N/A
		Known Expansion	00:19:48	01:19:10	9.55	23669	1
		STR	N/A	N/A	N/A	N/A	N/A
	Annotation	SNV/Indel	03:06:26	12:25:44	7.74	402697	71254
		SV + MEI	N/A	N/A	N/A	N/A	N/A
	Report Generation	SNV/Indel	01:24:58	05:39:51	0.02	1953279	8
		SV + MEI	N/A	N/A	N/A	N/A	N/A
**DNAscan2**	Analysis	SNV/Indel Calling	03:54:10	15:36:41	1.00	81794	9294
		SV Calling	33:32:53	133:53:30	5.70	1222044	55738
		MEI Calling	05:53:05	23:32:20	8.75	2710405	38612
		Known Expansion	00:19:48	01:19:10	9.55	23669	1
		STR	00:44:51	02:59:24	0.66	28505	30
	Annotation	SNV/Indel	03:00:48	12:03:12	6.78	353781	35076
		SV + MEI	00:47:15	03:08:58	13.54	8653	8386
	Report Generation	SNV/Indel	01:22:17	05:29:06	0.02	1840523	10
		SV + MEI	00:11:52	00:47:27	15.00	980	2331

Supplementary Table 4. Comparison of the average elapsed time of DNAscan and DNAscan2 for each step following alignment, using whole genomes of 10 Project MinE control samples. The steps were categorised by the main stage of the workflow (Analysis, Annotation, Report Generation). 'N/A' represents tasks that could not be performed in DNAscan due to lack of available software.

Gene Name	Chromosome	Sources of Identification			Reference
		Manual Review	***ALSoD***	***ClinVar***	
DNAJC7	17q21.2	✓			(Farhan *et al.*, 2019)
ERBB4	2q34	✓			(Takahashi *et al.*, 2013)
ANXA11	10q22.3		✓		(Smith *et al.*, 2017)
ARPP21	3p22.3		✓		(Cooper-Knock *et al.*, 2019)
ATXN1	6p22.3		✓		(Tazelaar *et al.*, 2020)
C21orf2	21q22.3		✓	✓	(van Rheenen *et al.*, 2016)
CAV1	7q31.2		✓		(Cooper-Knock *et al.*, 2020)
CAV2	7q31.2		✓		(Cooper-Knock *et al.*, 2020)
CCNF	16p13.3		✓	✓	(Williams *et al.*, 2016)
DNMT3A	2p23.3		✓		(Chestnut *et al.*, 2011)
DNMT3B	20q11.21		✓		(Chestnut *et al.*, 2011)
ENAH	1q42.12		✓		(Cirulli *et al.*, 2015)
EPHA3	3p11.1		✓		(Uyan *et al.*, 2013)
ERLIN1	10q24.31		✓	✓	(Tunca *et al.*, 2018)
GLT8D1	3p21.1		✓		(Cooper-Knock *et al.*, 2019)
GPX3	5q33.1		✓		(Benyamin *et al.*, 2017)
KIF5A	12q13.3		✓		(Nicolas *et al.*, 2018)
MOBP	3p22.1		✓	✓	(van Rheenen *et al.*, 2016)
NEFL	8p21.2		✓	✓	(Benatar *et al.*, 2018)
NEK1	4q33		✓		(Kenna *et al.*, 2016)
PGRN	17q21.31		✓		(Philips *et al.*, 2010)
PNPLA6	19p13.2		✓	✓	(Pensato *et al.*, 2020)
RFTN1	3p24.3		✓		(Zhai *et al.*, 2009)
SCFD1	14q12		✓	✓	(van Rheenen *et al.*, 2016)
TIA1	2p13.3		✓	✓	(Mackenzie *et al.*, 2017)
UBQLN1	9q21.32		✓		(Wang, et al., 2020)
VRK1	14q32.2		✓	✓	(Nguyen *et al.*, 2015)
CAPN14	2p23.1			✓	(Dekker *et al.*, 2019)
CHRNA3	15q25.1			✓	(Sabatelli *et al.*, 2009)
CHRNB4	15q25.1			✓	(Sabatelli *et al.*, 2009)
CNTF	11q12.1			✓	(Laaksovirta *et al.*, 2008)
CYLD	16q12.1			✓	(Dobson-Stone *et al.*, 2020)
DDX20	1p13.2			✓	(Cacciottolo *et al.*, 2019)
DYNC1H1	14q32.31			✓	(Scarlino *et al.*, 2020)
ELP3	8p21.1			✓	(Bento-Abreu *et al.*, 2018)
EWSR1	22q12.2			✓	(Couthouis *et al.*, 2012)
GARS1	7p14.3			✓	(Corcia *et al.*, 2019)
GLE1	9q34.11			✓	(Kaneb *et al.*, 2015)
MFN2	1p36.22			✓	(Wang *et al.*, 2018)
MPZ	1q23.3			✓	(Bisogni *et al.*, 2022)
NIPA1	15q11.2			✓	(Blauw *et al.*, 2012)

PLEKHG5	1p36.31		✓	(Gonzalez-Quereda *et al.*, 2021)
PON1	7q21.3		✓	(Verde *et al.*, 2019)
PON3	7q21.3		✓	(Saeed *et al.*, 2006)
SCN11A	3p22.2		✓	(Castoro *et al.*, 2018)
SLC52A3	20p13		✓	(Johnson *et al.*, 2012)
SYNE1	6q25.2		✓	(Naruse *et al.*, 2020)
TRPV4	12q24.11		✓	(Pensato *et al.*, 2020)
UNC13A	19p13.11		✓	(Diekstra *et al.*, 2012)

Supplementary Table 5. New additions and/or reclassifications in the ALSgeneScanner database. Breakdown of identified gene sources are as follows: 2 manual review, 16 ALSoD, 9 ALSoD and ClinVar, and 22 ClinVar.

References

Anaconda Software Distribution. Computer software. Vers 4.10.3. Anaconda, Mar. 2022. Web. https://anaconda.com

Babraham Bioinformatics - FastQC A Quality Control tool for High Throughput Sequence Data (no date). Available at: https://www.bioinformatics.babraham.ac.uk/projects/fastqc/ (Accessed: 9 September 2021).

Benatar, M. et al. (2018). Neurofilament light: A candidate biomarker of presymptomatic amyotrophic lateral sclerosis and phenoconversion. *Ann Neurol.*, 84(1), 130–139.

Bento-Abreu, A. et al. (2018). Elongator subunit 3 (ELP3) modifies ALS through tRNA modification. *Hum Mol Genet.*, 27(7), 1276–1289.

Benyamin, B. et al. (2017). Cross-ethnic meta-analysis identifies association of the GPX3-TNIP1 locus with amyotrophic lateral sclerosis. *Nat Commun.*, 8(1), 611.

Bisogni, G. et al. (2022). Thr124Met myelin protein zero mutation mimicking motor neuron disease. *Amyotroph Lateral Scler Frontotemporal Degener.* 23(3-4), 299-304.

Blauw, H.M. et al. (2012). NIPA1 polyalanine repeat expansions are associated with amyotrophic lateral sclerosis. *Hum Mol Genet*., 21(11), 2497–2502.

Bolognini, D. et al. (2020). VISOR: a versatile haplotype-aware structural variant simulator for short- and long-read sequencing. *Bioinformatics*, 36(4), 1267–1269.

Cacciottolo, R. et al. (2019). SMN complex member Gemin3 self-interacts and has a functional relationship with ALS-linked proteins TDP-43, FUS and Sod1. *Sci Rep.*, 9(1), 18666.

Castoro, R. et al. (2018). SCN11A Arg225Cys mutation causes nociceptive pain without detectable peripheral nerve pathology. *Neurol Genet.*, 4(4), e255.

Chen, X. et al. (2016). Manta: rapid detection of structural variants and indels for germline and cancer sequencing applications. *Bioinformatics.*, 32(8), 1220–1222.

Chestnut, B.A. et al. (2011). Epigenetic regulation of motor neuron cell death through DNA methylation. *J Neurosci.,* 31(46), 16619–16636.

Cirulli, E.T. et al. (2015). Exome sequencing in amyotrophic lateral sclerosis identifies risk genes and pathways. *Science.,* 347(6229), 1436–1441.

Cock, P.J.A. et al. (2009). Biopython: freely available Python tools for computational molecular biology and bioinformatics. *Bioinformatics.*, 25(11), 1422–1423.

Cooper-Knock, J. et al. (2019). Mutations in the Glycosyltransferase Domain of GLT8D1 Are Associated with Familial Amyotrophic Lateral Sclerosis. *Cell Rep.*, 26(9), 2298-2306.

Cooper-Knock, J. et al. (2020). Rare Variant Burden Analysis within Enhancers Identifies CAV1 as an ALS Risk Gene. *Cell Rep.*, 33(9), 108456.

Corcia, P. et al. (2019). Typical bulbar ALS can be linked to GARS mutation. *Amyotroph Lateral Scler Frontotemporal Degener.*, 20(3–4), 275–277.

Couthouis, J. et al. (2012). Evaluating the role of the FUS/TLS-related gene EWSR1 in amyotrophic lateral sclerosis. *Hum Mol Genet.*, 21(13), 2899–2911.

Danecek, P. et al. (2011). The variant call format and VCFtools. *Bioinformatics.*, 27(15), 2156–2158.

Dekker, A.M. et al. (2019). Exome array analysis of rare and low frequency variants in amyotrophic lateral sclerosis. *Sci Rep.*, 9(1), 5931.

Diekstra, F.P. et al. (2012). UNC13A is a modifier of survival in amyotrophic lateral sclerosis. *Neurobiol Aging.*, 33(3), 630.e3–8.

Dobson-Stone, C. et al. (2020). CYLD is a causative gene for frontotemporal dementia – amyotrophic lateral sclerosis. *Brain.*, 143(3), 783–799.

Dolzhenko, E. et al. (2019). ExpansionHunter: a sequence-graph-based tool to analyze variation in short tandem repeat regions. *Bioinformatics.*, 35(22), 4754–4756.

Dolzhenko, E. et al. (2020).ExpansionHunter Denovo: a computational method for locating known and novel repeat expansions in short-read sequencing data. *Genome Biol.*, 21(1), 102.

Ewels, P. et al. (2016). MultiQC: summarize analysis results for multiple tools and samples in a single report. *Bioinformatics.*, 32(19), 3047–3048.

Farhan, S.M.K. et al. (2019). Exome sequencing in amyotrophic lateral sclerosis implicates a novel gene, DNAJC7 encoding a heat-shock protein. *Nat Neurosci.*, 22(12), 1966–1974.

Faust, G.G. and Hall, I.M. (2014). SAMBLASTER: fast duplicate marking and structural variant read extraction. *Bioinformatics.,* 30(17), 2503–2505.

Fazal, S. et al. (2020). Large scale in silico characterization of repeat expansion variation in human genomes. *Sci Data.*, 7(1), 294.

Gardner, E.J. et al. (2017). The Mobile Element Locator Tool (MELT): population-scale mobile element discovery and biology. *Genome Res.*, 27(11), 1916–1929.

Geoffroy, V. et al. (2018). AnnotSV: an integrated tool for structural variations annotation. *Bioinformatics.*, 34(20), 3572–3574.

Geoffroy, V. et al. (2021). AnnotSV and knotAnnotSV: a web server for human structural variations annotations, ranking and analysis. *Nucleic Acids Res.*, 49(W1), W21–W28.

Gonzalez-Quereda, L. et al. (2021). Novel PLEKHG5 mutations in a patient with childhood-onset lower motor neuron disease. *Ann Clin Transl Neurol.*, 8(1), 294–299.

Haplotype Comparison Tools (2021). Illumina. Available at: https://github.com/Illumina/hap.py (Accessed: 29 September 2021).

Jeffares, D.C. et al. (2017). Transient structural variations have strong effects on quantitative traits and reproductive isolation in fission yeast. *Nat Commun.*, 8, 14061.

Johnson, J.O. et al. (2012). Exome sequencing reveals riboflavin transporter mutations as a cause of motor neuron disease. *Brain.*, 135(9), 2875–2882.

Kaneb, H.M. et al. (2015). Deleterious mutations in the essential mRNA metabolism factor, hGle1, in amyotrophic lateral sclerosis. *Hum Mol Genet.*, 24(5), 1363–1373.

Kenna, K.P. et al. (2016). NEK1 variants confer susceptibility to amyotrophic lateral sclerosis. *Nat Genet.*, 48(9), pp. 1037–1042.

Kim, D. et al. (2019). Graph-based genome alignment and genotyping with HISAT2 and HISAT-genotype. *Nat Biotechnol.*, 37(8), 907–915.

Kim, S. et al. (2018). Strelka2: fast and accurate calling of germline and somatic variants. *Nat Methods.*, 15(8), 591–594.

Kosugi, S. et al. (2019). Comprehensive evaluation of structural variation detection algorithms for whole genome sequencing. *Genome Biol.*, 20(1), 117.

Li, H. et al. (2009). The Sequence Alignment/Map format and SAMtools. *Bioinformatics.,* 25(16), 2078–2079.

Li, H. (2011). A statistical framework for SNP calling, mutation discovery, association mapping and population genetical parameter estimation from sequencing data. *Bioinformatics.*, 27(21), 2987–2993.

Li, H. (2013). Aligning sequence reads, clone sequences and assembly contigs with BWA-MEM. *ArXiv.,* 1303.3997 [q-bio].

Mackenzie, I.R. et al. (2017). TIA1 Mutations in Amyotrophic Lateral Sclerosis and Frontotemporal Dementia Promote Phase Separation and Alter Stress Granule Dynamics. *Neuron.*, 95(4), 808-816.e9.

Mölder, F. et al. (2021). Sustainable data analysis with Snakemake. *F1000Res.*,10,33.

Naruse, H. et al. (2020). Juvenile amyotrophic lateral sclerosis with complex phenotypes associated with novel SYNE1 mutations. *Amyotroph Lateral Scler Frontotemporal Degener.*, 1–3.

Nguyen, T.P. et al. (2015). Expanding Phenotype of VRK1 Mutations in Motor Neuron Disease. *J Clin Neuromuscul Dis.*, 17(2), 69–71.

Nicolas, A. et al. (2018). Genome-wide Analyses Identify KIF5A as a Novel ALS Gene. *Neuron.*, 97(6), 1268-1283.e6.

Pensato, V. et al. (2020). Sorting Rare ALS Genetic Variants by Targeted Re-Sequencing Panel in Italian Patients: OPTN, VCP, and SQSTM1 Variants Account for 3% of Rare Genetic Forms. *J Clin Med.*, 9(2), 412.

Philips, T. et al. (2010). Microglial upregulation of progranulin as a marker of motor neuron degeneration. *J Neuropathol Exp Neurol.*, 69(12), 1191–1200.

Project MinE ALS Sequencing Consortium (2018). Project MinE: study design and pilot analyses of a large-scale whole-genome sequencing study in amyotrophic lateral sclerosis. *Eur J Hum Genet.,* 26(10), 1537–1546.

Pysam (2021). pysam-developers. Available at: https://github.com/pysam-developers/pysam (Accessed: 9 September 2021).

PySimpleGUI (no date). Available at: https://pysimplegui.readthedocs.io/en/latest/ (Accessed: 9 September 2021).

Quinlan, A.R. and Hall, I.M. (2010). BEDTools: a flexible suite of utilities for comparing genomic features. *Bioinformatics.*, 26(6), 841–842.

Rafehi, H. et al. (2019). Bioinformatics-Based Identification of Expanded Repeats: A Non-reference Intronic Pentamer Expansion in RFC1 Causes CANVAS. *Am J Hum Genet.*, 105(1), 151–165.

Rausch, T. et al. (2012). DELLY: structural variant discovery by integrated paired-end and split-read analysis. *Bioinformatics.,* 28(18), i333–i339.

van Rheenen, W. et al. (2016). Genome-wide association analyses identify new risk variants and the genetic architecture of amyotrophic lateral sclerosis. *Nat Genet.* 48(9), 1043–1048.

Sabatelli, M. et al. (2009). Rare missense variants of neuronal nicotinic acetylcholine receptor altering receptor function are associated with sporadic amyotrophic lateral sclerosis. *Hum Mol Genet.*, 18(20), 3997–4006.

Saeed, M. et al. (2006). Paraoxonase cluster polymorphisms are associated with sporadic ALS. *Neurology.*, 67(5), 771–776.

Scarlino, S. et al. (2020). Burden of Rare Variants in ALS and Axonal Hereditary Neuropathy Genes Influence Survival in ALS: Insights from a Next Generation Sequencing Study of an Italian ALS Cohort. *Int J Mol Sci.*, 21(9), 3346.

Laaksovirta, H. et al. (2008). Serum level of CNTF is elevated in patients with amyotrophic lateral sclerosis and correlates with site of disease onset. *Eur J Neurol.*, 15(4), 355-359.

Smith, B.N. et al. (2017). Mutations in the vesicular trafficking protein annexin A11 are associated with amyotrophic lateral sclerosis. *Sci Transl Med.*, 9(388), eaad9157.

spiralgenetics/truvari (2021). Spiral Genetics. Available at: https://github.com/spiralgenetics/truvari (Accessed: 10 September 2021).

Sudmant, P.H. et al. (2015). An integrated map of structural variation in 2,504 human genomes. *Nature.*, 526(7571), 75–81.

Takahashi, Y. et al. (2013). ERBB4 mutations that disrupt the neuregulin-ErbB4 pathway cause amyotrophic lateral sclerosis type 19. *Am J Hum Genet*, 93(5), 900–905.

Tarasov, A. et al. (2015). Sambamba: fast processing of NGS alignment formats. *Bioinformatics.*, 31(12), 2032–2034.

Tazelaar, G.H.P. et al. (2020). ATXN1 repeat expansions confer risk for amyotrophic lateral sclerosis and contribute to TDP-43 mislocalization. *Brain Commun.*, 2(2), fcaa064.

Tunca, C. et al. (2018). ERLIN1 mutations cause teenage-onset slowly progressive ALS in a large Turkish pedigree. *Eur J Hum Genet.*, 26(5), 745–748.

Uyan, Ö. et al. (2013). Genome-Wide Copy Number Variation in Sporadic Amyotrophic Lateral Sclerosis in the Turkish Population: Deletion of EPHA3 Is a Possible Protective Factor. *PLoS ONE.*, 8(8), e72381.

Vendrell-Mir, P. et al. (2019). A benchmark of transposon insertion detection tools using real data. *Mob DNA*, 10(1), 53.

Verde, F. et al. (2019). PON1 is a disease modifier gene in amyotrophic lateral sclerosis: association of the Q192R polymorphism with bulbar onset and reduced survival. *Neurol Sci.,* 40(7), 1469–1473.

Wang, K. et al. (2010). ANNOVAR: functional annotation of genetic variants from high-throughput sequencing data. *Nucleic Acids Res.*, 38(16), e164–e164.

Wang, L. et al. (2018). Mitofusin 2 Regulates Axonal Transport of Calpastatin to Prevent Neuromuscular Synaptic Elimination in Skeletal Muscles. *Cell Metab.,* 28(3), 400-414.e8.

Wang, S. et al. (2020). Overexpression of UBQLN1 reduces neuropathology in the P497S UBQLN2 mouse model of ALS/FTD. *Acta Neuropathol Commun.*, 8(1), 164.

Williams, K.L. et al. (2016). CCNF mutations in amyotrophic lateral sclerosis and frontotemporal dementia. *Nat Commun.*, 7, 11253.

Zhai, J. et al. (2009). Proteomic Characterization of Lipid Raft Proteins in ALS Mouse Spinal Cord. *FEBS J.*, 276(12), 3308–3323.
